# Degradable Polymer Stars Based on Tannic Acid Cores by ATRP

**DOI:** 10.3390/polym11050752

**Published:** 2019-04-28

**Authors:** Julia Cuthbert, Saigopalakrishna S. Yerneni, Mingkang Sun, Travis Fu, Krzysztof Matyjaszewski

**Affiliations:** 1 Department of Chemistry, Carnegie Mellon University, 4400 Fifth Avenue, Pittsburgh, PA 15213, USA; jcuthber@andrew.cmu.edu (J.C.); mingkang@cmu.edu (M.S.); travisfu@cmu.edu (T.F.); 2 Department of Biomedical Engineering, Carnegie Mellon University, 5000 Forbes Avenue, Pittsburgh, PA 15213, USA; syerneni@andrew.cmu.edu

**Keywords:** degradable polymer, tannic acid, ATRP, polymer star

## Abstract

Degradable polymers are crucial in order to reduce plastic environmental pollution and waste accumulation. In this paper, a natural product, tannic acid was modified to be used as a polymer star core. The tannic acid was modified with atom transfer radical polymerization (ATRP) initiators and characterized by ^1^H NMR, FT-IR, and XPS. Twenty-five arm polymer stars were prepared by photoinduced ATRP of poly(methyl methacrylate) (PMMA) or poly(oligo(ethylene oxide) methacrylate) (molar mass M_w_ = 300 g/mol) (P(OEO_300_MA)). The polymer stars were degraded by cleaving the polymer star arms attached to the core by phenolic esters under mild basic conditions. The stars were analyzed before and after degradation by gel permeation chromatography (GPC). Cytotoxicity assays were performed on the P(OEO_300_MA) stars and corresponding degraded polymers, and were found to be nontoxic at the concentrations tested.

## 1. Introduction

**Scheme 1:**
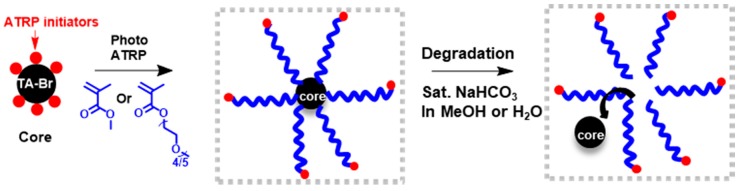
The synthesis of the polymer stars from a modified tannic acid core (TA-Br) by photo atom transfer radical polymerization (ATRP) and the subsequent degradation (cleaving the polymer arms) using a solution of sodium bicarbonate and either methanol or water. Note: For an easily readable illustration, only six polymer star arms are shown instead of twenty-five.

Synthetic polymers have become indispensable to modern life, from commodity materials, such as packaging, to medicine and aerospace. Unfortunately, many traditional synthetic, commercial polymers are not biodegradable, which results in waste disposal problems and pollution [1]. For this reason, green polymer chemistry has been steadily gaining interest over the past several decades [2]. One strategy in green chemistry is to design degradable materials, which should minimize waste and facilitate recycling [2]. Thus, we aimed to use a natural product, tannic acid (TA), as a core for degradable polymer stars (Scheme 1).

Tannic acid is a polyphenol found in plants, which has a molar mass of 1701.19 (g/mol) and a chemical formula of C_76_H_52_O_46_ [3]. It is an inexpensive, abundant, and commercially available product approved by the United States Federal Drug Administration (FDA). Moreover, TA is also biodegradable [4,5,6,7], and exhibits anti-inflammatory and antimicrobial properties [8,9,10]. Given these excellent properties, TA has attracted the attention of various research groups. For example, TA/iron(III) complexes were used as a cytoprotective, degradable coating for mammalian cell cultures [11]. In recent years, TA has been applied to polymeric materials. Poly(tannic acid) has been reported as using multifunctional epoxides in the presence of base to crosslink TA [12]. In addition, polymer composites containing poly(ethylene glycol) (PEG) and TA had excellent mucoadhesive properties at neutral pH (esophageal conditions), but hydrolyzed rapidly in stomach acid conditions (pH = 1.2) [4]. TA has also been used as a crosslinking agent for hydrogels via hydrogen bonding between the hydroxy groups and the polymer [13,14,15,16].

Pure tannic acid has 25 hydroxy groups potentially available for chemical bonding and modifications. Its structure lends itself to functioning as a polymer star core. A polymer star consists of multiple linear polymers (arms) attached at one point (the core) [17,18,19,20]. Therefore, the tannic acid hydroxy groups can be substituted with a new functional group and subsequently grow polymer arms from the core (core-first method). There have been successful reports of functionalizing TA and then growing poly(aniline) from the core by chemical oxidation polymerization to form conductive stars (core-first method) [21,22]. Polyurethanes have also been synthesized and then attached to the tannic acid core (arm-first method) [23]. Most recently, polymers have been grafted from TA by reversible addition fragmentation chain transfer (RAFT) polymerization and click chemistry [24].

**Scheme 2:**
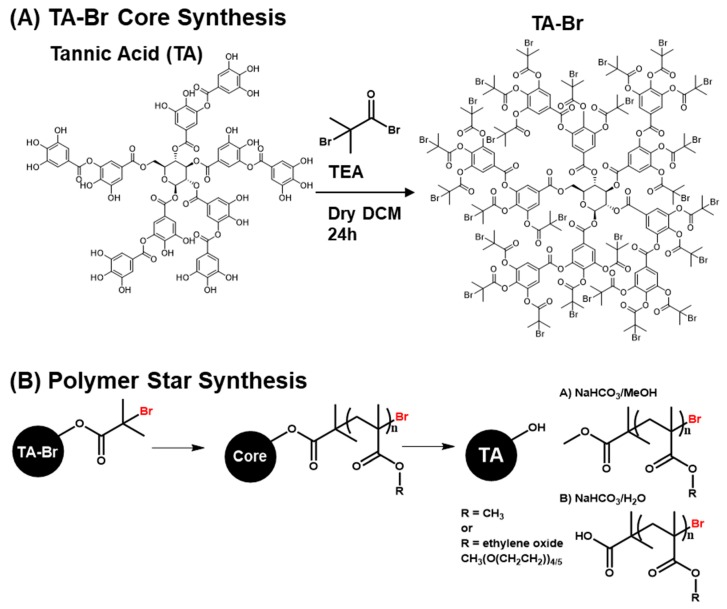
(**A**) The synthesis of the polymer star core from tannic acid. (**B**) Focusing on a single ATRP initiator, the polymers star synthesis and subsequent degradation (cleaving the polymer arms) using in the presence sodium bicarbonate and either methanol or water.

In this paper, tannic acid was fully functionalized with α-bromoisobutyryl bromide (Br-iBBr) by a nucleophilic substitution reaction, to produce TA-Br (Scheme 1 and Scheme 2), for subsequent polymerization by atom transfer radical polymerization (ATRP) [25,26,27,28]. TA functionalized with two eq. isobutyryl bromide ATRP initiators has been used as a surface initiator in order to graft antifouling and antimicrobial polymers from a variety of substrates [9]. TA has also been used in combination with ATRP to prepare well defined nanotubes [29]. Other polyphenols, such as quercetin, have been used as ATRP star cores [3,30]. However, to the best of our knowledge, there are no reports of tannic acid ATRP initiators for star polymer architecture. In addition, the ATRP initiators were attached to the TA core by phenolic esters, which can be cleaved from the core via a transesterification using a mild base (sodium bicarbonate) in either methanol or water. Thus, we report the synthesis and characterization of tannic acid ATRP star cores (TA-Br) for biocompatible and degradable stars.

## 2. Materials and Methods 

### 2.1. Materials

Methyl methacrylate (MMA, 99%, Sigma-Aldrich, St. Louis, MO, USA) and oligo(ethylene glycol) methyl ether methacrylate (OEO_300_MA, average molecular weight 300, Sigma-Aldrich) were passed through basic alumina column to remove radical inhibitors prior to use. 1-Methyl-2-pyrrolidinone (NMP, ≥99.0%, Sigma-Aldrich) was sparged with nitrogen gas and stirred in a sealed round bottom flask containing molecular sieves (beads, Sigma-Aldrich, 4 Å, 4-8 mesh) overnight prior to use. Acetonitrile (ACN, Sigma-Aldrich), α-bromoisobutyryl bromide (Br-iBBr, Sigma-Aldrich, 98%), dichloromethane (DCM, ACS grade, Fisher Scientific, Hampton, VA, USA), copper(II) bromide (CuBr_2_, 99%, Sigma-Aldrich), N,N-dimethylformamide (DMF, ACS grade, Fisher Scientific), dimethyl sulfoxide (DMSO, ACS grade, Fisher Scientific), deuterated DMSO (DMSO-d_6_, 99.9%, Cambridge Isotope Laboratories, Tewksbury, MA, USA), hexane (GR ACS, EMD Millipore Corporation, Burlington, VT, USA), ethyl acetate (ACS grade, Fisher Scientific), magnesium sulfate anhydrous (MgSO_4_, certified ACS, Fisher Chemical, Hampton, VA, USA) methanol (MeOH, GR ACS, EMD Millipore Corporation), sodium bicarbonate (certified ACS, Fisher Chemical), sodium chloride (NaCl, certified ACS, Fisher Chemical), tannic acid (ACS reagent, Sigma-Aldrich), tetrahydrofuran (THF, GR ACS, EMD Millipore Corporation), triethylamine (TEA, ≥99%, Sigma-Aldrich), 2,3,6,7,10,11-triphenylenehexol (TP-OH, TCI Chemicals, 95%), and tris[2-(dimethylamino) ethyl]amine (Me_6_TREN, KOEI Chemical Co., LTD, Tokyo, Japan) were used as received.

### 2.2. Material Characterization

The number averaged molecular weight (M_n_) and molecular weight distribution (Ð, M_w_/M_n_) were determined by gel permeation chromatography (GPC) with either DMF or THF as the eluent. DMF GPC analysis with different polymer samples was conducted with an Agilent 1260 Infinity II pump and Wyatt Optilab T-rEX RI detector using PSS GRAM analytical column (10 µm particle size) in HPLC grade DMF as an eluent at 50 °C and at a flow rate of 1 mL min^−1^. The THF as eluent GPC system used a Waters 515 HPLC pump and a Waters 2414 refractive index detector using PSS columns (SDV 102, 103, 105 Å) with a flow rate of 1 mL/min at 35°C. Linear poly(methyl methacrylate) (PMMA) standards were used for both GPC calibrations and the GPC results analyzed in WinGPC UniChrom software from PSS.

### 2.3. Instrumentation

Nuclear Magnetic Resonance (^1^H NMR) was carried out with Bruker Ultrashield 500 MHz operating at 500 MHz for ^1^H NMR DMSO-d_6_ as the solvent. Photo-induced polymerization was carried out using a MelodySusie^®^ UV lamp. The measured power at λ = 365 nm was 5.3 mW/cm^2^. Fourier-transform infrared spectroscopy (FT-IR) was carried out on a PerkinElmer Frontier FT-IR spectrometer and the data was processed using PerkinElmer Spectrum software, version 10.4.4. UV/vis spectroscopy was performed using an Agilent Technologies Cary 60 UV-Vis and the data was collected with Agilent Technologies Cary WinUV scan application (Version 5.1.0.1016). X-Ray photoelectron spectroscopy (XPS) experiments were performed using a Thermo Scientific ESCALAB 250Xi X-ray Photoelectron Spectrometer Microprobe. X-ray spot size was selected to 900 µm. The flood electron source was used for charge compensation.

### 2.4. TA-Br Synthesis

Tannic acid (1701.19 g/mol, 1.76 mmol, 3.00 g, 1 eq.), TEA (57.4 mmol, 8.0 mL, 33 eq.), and dry NMP (100 mL) were added to a dry 250 mL round bottom flask containing a stir bar, capped, and stirred in an ice bath under nitrogen gas. Using a syringe pump, α-Br-iBBr (48.5 mmol, 6.0 mL, 28 eq.) was added dropwise over 1 h. The solution was removed from the ice bath and stirred in a sealed flask for 3 days at room temperature. A white precipitate (amine salt) formed during this time. After 3 days, ethyl acetate (15 mL), was added. The solution was gravity filtered to remove the precipitated salt. The solution was then added to 1kD RCD dialysis tubing and dialyzed against DI water for 5 cycles over 48 h. The product, TA-Br, was insoluble in water and a tan colored precipitate was observed in the dialysis tubing. The product was collected, dissolved in DCM (50 mL), and washed with 2× saturated NaHCO_3_ in DI water solution (20 mL), 1× DI water (20 mL), 1× brine (20 mL), then dried over MgSO_4_, and concentrated in vacuo. The TA-Br was finally precipitated into cold hexane. Yield: 4.19 g, 43.6%. M_w,theo_ TA-Br = 5425.69 g/mol. The final compound was analyzed by ^1^H-NMR: (500 MHz, DMSO-d) δ 8.50–7.40 (m, 19H), 6.53 (s, 0.9H), 6.30 (s, 1H), 5.75 (s, 2.2H), 4.82 (s, 1H), 4.65–4.58 (d, 2.3H), 2.04 (s, 113H), 1.92–1.86 (t, 31H).

### 2.5. General Polymer Star Synthesis

The general experimental procedure for the polymer stars is reported here. For details, refer to the supporting information (Table S1). To a Schlenk flask, TA-Br, monomer (MMA or OEO_300_MA), solvent (DMSO or anisole), and a stock solution containing CuBr_2_/Me_6_TREN (1:6 molar eq.; CuBr_2_ = 6 mg/mL and Me_6_TREN = 39 mg/mL) in DMF was added. [Monomer]/TA-Br/CuBr_2_/Me_6_TREN = target DP/1/1/6. The flask was covered in aluminum foil and sparged with N_2_ gas for 30 min. The polymerization was performed by photoinduced ATRP using a MelodySusie UV lamp (λ_max_ = 365 nm; 5.2 mW/cm^2^). The reactions were followed by ^1^H NMR. The polymerizations were stopped by removal of UV light and exposure to ambient oxygen.

### 2.6. Degradation Procedure

The polymer stars were measured in a 20 mL glass vial containing a stir bar. To the vial, a saturated suspension NaHCO_3_ in MeOH (TA-PMMA stars) or in water (TA-P(OEO_300_MA) stars) was added so that the concentration of polymer star in solution was 3 mg/mL. The solutions were stirred for 72 h. The transesterification was stopped by vacuum evaporation of the solvent.

### 2.7. Cell Culture

Human embryonic kidney cell line (HEK293), murine embryonic fibroblasts cell line (NIH3T3) and human keratinocyte cell line (HaCaT) were grown and maintained in Dulbecco’s modified eagle media (DMEM; Gibco, Gaithersburg, MD, USA) supplemented with 10 % fetal bovine serum (FBS, Thermo Fisher Scientific, Waltham, MA, USA) and 1 % Penicillin-streptomycin (Gibco, Gaithersburg, MD, USA).

### 2.8. Cytotoxicity Assay

Cytotoxicity was assessed using a direct CyQUANT^®^ nucleic acid-sensitive fluorescence assay (Thermo Fisher Scientific, Waltham, MA, USA) according to the manufacturer’s instructions. Briefly, 100 µL aliquots of cell suspension containing 1.25 × 103 cells/mL were plated in wells of a 96-well microplate (Corning Inc., Corning, NY, USA) and allowed to adhere for 6h. Treatments with varying concentrations of polymer/degraded polymer were added to respective treatment wells and co-incubated with cells for 72h. As a cytotoxicity control, 5 µg/mL Saponin (#84510, Sigma-Aldrich) was used. Next, cells were labeled with CyQUANT^®^ Direct and fluorescence intensities were measured with TECAN spectrophotometer reader (TECAN, Männedorf, Switzerland). Cytotoxicity was assessed by normalizing fluorescence intensities to control (no treatment) group and plotted as percent viability.

## 3. Results and Discussion

### 3.1. Synthesis and Characterization of TA-Br

Nucleophilic acyl substitution was employed to synthesize the phenolic ester ATRP star core based on tannic acid (TA-Br) (Scheme 2). The phenol groups were substituted for α-bromoisobutyrate (iBBr) ATRP initiator sites using α-bromo-isobutyryl bromide and triethylamine (TEA) as the base (Scheme 2). As the objective was to completely substitute the phenol groups to produce a core with 25 iBBr sites, a slight excess of Br-iBBr (28 eq.) was used.

**Figure 1:**
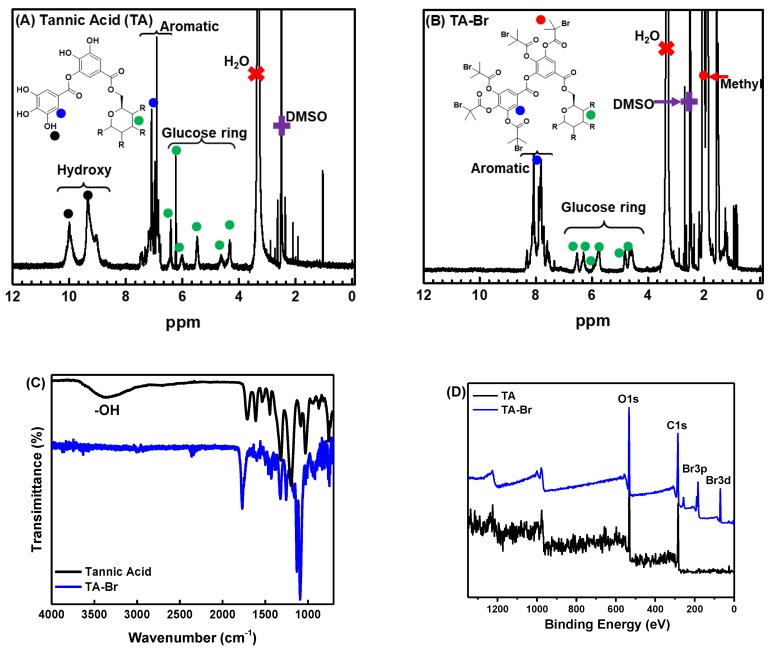
(**A**) The ^1^H NMR spectra of tannic acid (TA) and (**B**) TA-Br with the peaks identified. For clarity, only one polyphenol arm of TA and TA-Br is shown (R = the 4 other identical phenol branches) and representative peaks are shown with colored circles. (**C**) FT-IR spectra of TA (black) and TA-Br (blue). (**D**) XPS survey spectra of TA (black) and TA-Br (blue).

The TA-Br product obtained was first characterized by ^1^H NMR in DMSO-d_6_ and the results support the proposed structure of TA-Br (Scheme 2A). The phenol hydroxy peaks are not present in the TA-Br NMR and the aromatic peaks shifted (Figure 1A,B), indicating that all the phenol groups were substituted with iBBr. The central glucose ring peaks (7 protons) were still present (Figure 1B, green circles) and the iBBr methyl protons also were present (Figure 1B, red circle). This agrees with similar ^1^H NMR spectra reported in the literature [4,9]. The TA-Br ^1^H NMR integration values ratios were as follows: aromatic/glucose/methyl = 19H/7H/144H (Section 2.4 TA-Br Synthesis). For a natural product, this was in excellent agreement with the theoretical values = 20H/7H/150H. In addition, the Fourier Transform Infrared (FT-IR) spectroscopy corroborated the disappearance of the hydroxy groups and showed increased ester stretches (Figure 1C) and the UV/vis spectra of TA and ‘TA-Br were distinct (Figure S1). The TA and TA-Br were further characterized by X-Ray Photoelectron Spectroscopy (XPS). The survey scan of TA-Br revealed the presence of Br 3p and Br 3d lines, which were not observed in the TA scan (Figure 1D). Both samples were further scanned for Br 3d and this peak was observed in the TA-Br sample, but not the TA sample (Figure S2).

### 3.2. Synthesis of Polymer Stars by Photo ATRP

Having confirmed that the tannic acid was functionalized with ATRP initiator sites, the next step was to polymerize methacrylate monomers to form star arms from the TA-Br Core by photo ATRP (Table 1, Table S1). Polymer stars were synthesized using either poly(methyl methacrylate) (PMMA) or poly(oligo ethylene oxide methacrylate) (M_w_ = 300 g/mol) (P(OEO_300_MA)) targeting two different degrees of polymerization (DP) (Table 1 and Table S1). PMMA polymer arms were grown in order to have the best possible comparison with gel permeation chromatography (GPC) calibration standards. P(OEO_300_MA) was used because poly(ethylene oxide/glycol) (PEO or PEG) is considered non-toxic and biocompatible. Of the four polymer stars synthesized (Table 1, entries 1, 3, 5, and 7), three had dispersity (*Ð*) < 1.3, indicating controlled radical polymerizations. Entry 7 had a higher *Ð* of 1.54, which was attributed to the fact that it was a macromonomer being polymerized from a sterically hindered core with the highest DP of the samples. It should be noted that the polymer star M_n,theo_ and M_n,GPC_ were significantly different due to the difference in hydrodynamic volume between a polymer star and a linear PMMA polymer standard (Table 1, entries 1, 3, 5, and 7).

**Figure 2:**
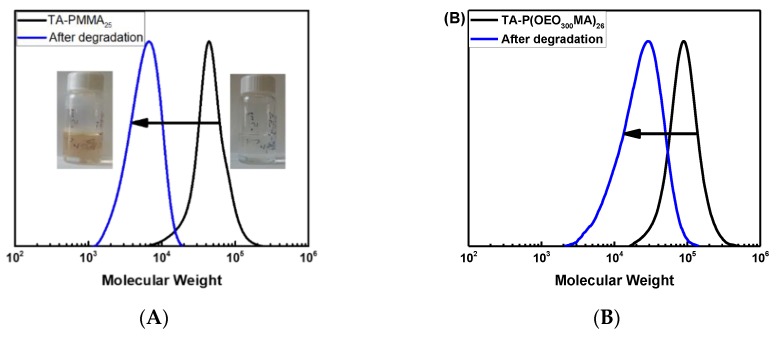

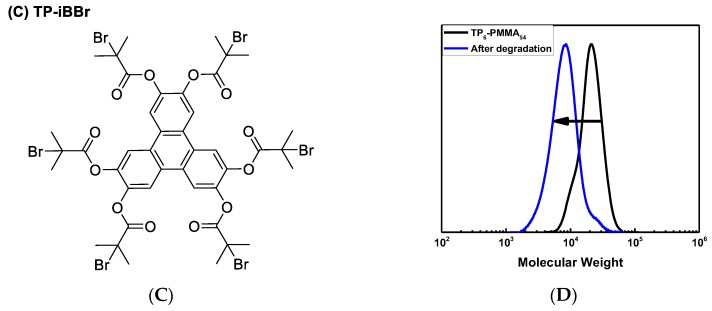
(**A**) The GPC traces of tannic acid-poly(methyl methacrylate) (TA-PMMA) PMMA_25_ and (**B**) TA-P(OEO_300_MA)_25_ before (black) and after (blue) degradation. Insets in A show the solution color before and after degradation. (**C**) The structure of the TP_6_Br core. (**D**) The GPC traces of TP-PMMA_54_ before (black) and after (blue) degradation.

Since the polymer star arms were attached to the TA core by phenolic ester bonds, the arms could be cleaved by a saponification reaction in the presence of a base. The polymer stars were placed in solutions of sodium bicarbonate (NaHCO_3_) suspension in either methanol (MeOH) for TA-PMMA or water (H_2_O) for TA-P(OEO_300_MA) for 72 h. As the degradation progressed, the solutions turned a tan color (Figure 2A), which indicated the presence of free tannins. The cleaved PMMA polymer arms were characterized by GPC (Table 1 entries 2 and 4, Figure 2A and Figure S3A). The result was a clear decrease in molecular weight (Figure 2A), confirming that the polymer arms had been removed from the core. In addition, the photo ATRP had produced well-controlled chains with *Ð* < 1.3. However, the M_n,GPC_ was either 3×(TA-PMMA_7_) or 2× (TA-PMMA_25_) greater than the M_n,theo_. Given that the TA-Br is such a sterically hindered structure, it was expected that the 10 “inner sphere” iBBr sites would be difficult to access. The initiation efficiency (*f* = M_n, theo_/ M_n, GPC_) of these sites was likely low or zero. The total core *f* was between 32–53% (Table 1). Similar results were observed when degrading the P(OEO_300_MA) stars (Table 1 entries 6 and 8, Figure 2B and Figure S3B). Although the M_n,GPC_ significantly decreased after degradation, unlike the TA-PMMA stars, the *Ð* increased. In contrast to MMA, in this system, the OEO_300_MA was not as well controlled by ATRP. The OEO_300_MA monomer has more bulky or longer side chains than PMMA, which would increase steric hinderance between growing polymer chains and further decrease initiation efficiency from the TA-Br. During polymerization, it also possible that higher DP chains shielded lower DP chains, and this also contributed to the higher *Ð*.

In order to further understand the TA based polymer stars, well-defined, small molecule star core, tetraphenyl-bromoisobutyrate (TP-iBBr), was prepared (Scheme S1, Figure 2C). A TP_6_-PMMA_54_ star was synthesized (M_n, theo_ = 3.13 × 10^4^, M_n, GPC_ = 2.69 × 10^4^, *Ð* = 1.09) and then degraded in a saturated solution of NaHCO_3_ in MeOH (Figure 2D). The polymer arms were analyzed by GPC: M_n, theo_ = 5.59 × 10^3^, M_n, GPC_ = 7.08 × 10^3^, *Ð* = 1.26 (Figure 2D). In comparison to the TA-PMMA stars, which degraded in 24 h, the TP-PMMA_54_ took approximately 12 days to degrade. This could be due to two reasons: (1) the insolubility of PMMA in MeOH and (2) the fact that the 2,3,6,7,10,11-Triphenylenehexol (TP-OH, liberated core) would be stable in MeOH. The only phenolic esters are attached to the tetraphenyl core and are the only functional groups susceptible to nucleophilic attack. In comparison, the TA core is comprised of phenolic ester linkages, which are hydrolysable. Therefore, TA can be completely broken down into smaller molecules. Indeed, its biodegradation pathways have been studied [5,31], and its thermal degradation [32]. Degradation of TA-P(OEO_300_MA)_25_ was followed using by GPC and UV/vis (Figure S4A,B). As the reaction was followed by UV/vis spectroscopy (Figure S4B), a steady increase in absorbance was observed at 315 nm, suggesting the liberation of the TA core (Figure S1). But in addition, increased absorbance was observed around 400 nm, which were not observed when analyzing pure TA or TA-Br (Figure S1). Therefore, these were likely from other tannin molecules.

### 3.3. Cytotoxicity

**Figure 3:**
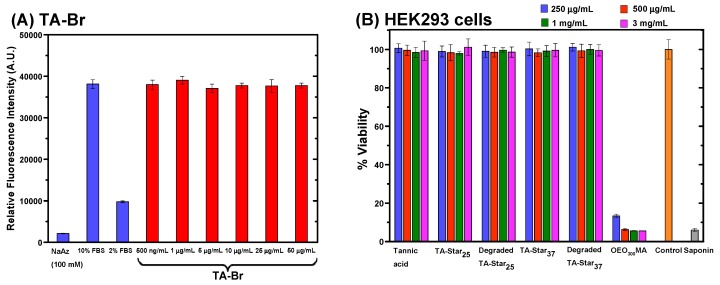
The cytotoxicity assays performed using HEK293 cells. (**A**) Assays of TA-Br at six concentrations (red) and the positive (FBS) and negative (NaAz) controls vs the relative fluorescence intensity. (**B**) The percent viability after 72 h of TA-P(OEO_300_MA) polymer stars, samples after degradation, and OEO_300_MA monomer at four concentrations from 250μg-3 mg/mL. Positive (orange) and negative controls (Saponin, grey) are also shown.

Much of the motivation for using a natural product based polymer star cores, was to synthesize biocompatible polymeric materials. For that reason, cytotoxicity assays were formed on TA-Br, TA, and the TA-P(OEO_300_MA) stars (Figure 3A,B). The TA-Br itself was assessed at concentrations from 500 ng/mL to 50 μg/mL (Figure 3A, red). For a comparison, 100 mM sodium azide (NaN_3_) was used as a negative control and two solutions of fetal bovine serum (FBS at 2% and 10%) were used as positive controls (Figure 3A, blue). After 72 h, at all concentrations tested, the TA-Br showed a similar florescence intensity as the positive 10% FBS control, indicating good cell viability.

Next, the polymer stars were tested using three distinct types of cells: human embryonic kidney cell line (HEK293) (Figure 3B), murine embryonic fibroblasts cell line (NIH3T3) (Figure S5A), and human keratinocyte cell line (HaCaT) (Figure S5B). Tannic acid, OEO_300_MA monomer, and the TA-P(OEO_300_MA) stars and degraded solutions (saturated NaHCO_3_ in water) were tests at four concentrations between 250 μg/mL to 3 mg/mL. As anticipated, the OEO_300_MA was cytotoxic, whereas the TA, polymer stars, and degraded solutions showed approximately 100% cell viability after 72 h.

## 4. Conclusions

A polymer star core has been synthesized from a natural product, tannic acid. Tannic acid was functionalizing tannic acid with ATRP initiator sites, for subsequent growth of polymer arms by photo ATRP. These polymer stars were degradable under mild basic conditions. Finally, cytotoxicity assays on the TA-Br, TA-P(OEO_300_MA) stars, and the degraded versions were found to be nontoxic.

## Figures and Tables

**Table 1:** The polymer stars prepared and degraded in either methanol/bicarb (PMMA) or water/bicarb (P(OEO_300_MA)).

Entry ^1^	TA-Polymer Star	Arm DP_theo_^2^	M_n, theo_ × 10^−3^	M_n, GPC_ × 10^−3^	*Ð*	*f ^3^*
1	TA-PMMA_7_	7	23.6	11.1	1.23	-
2	Polymer arms post degradation	7	0.905	2.86	1.18	32%
3	TA-PMMA_25_	25	67.5	39.4	1.26	-
4	Polymer arms post degradation	25	2.66	5.03	1.26	53%
5	TA-P(OEO_300_MA)_25_	25	200	78.1	1.26	-
6	Polymer arms post degradation	25	7.98	17.6	1.56	45%
7	TA-P(OEO_300_MA)_37_	37	287	125	1.54	-
8	Polymer arms post degradation	37	11.4	32.7	1.84	35%

^1^ Conditions: MMA/TA-Br/CuBr_2_/Me_6_TREN = target DP/1/1/6; [MMA] = 0.9M; Solv. = DMSO by photo ATRP (λ_max_ = 365 nm; 5.2 mW/cm^2^). ^2^ Determined by ^1^H NMR conversion and assuming 25 arms per Star. ^3^For PMMA, THF GPC and for P(OEO_300_MA) DMF GPC; linear PMMA Standards. ^3^ Initialtion efficiency (*f*) = M_n, theo_/M_n, GPC_ (report in percentage) and calculated using the M_n,theo_ per polymer arm and the observed M_n, GPC_.

## Supplementary Materials

The following are available online at https://www.mdpi.com/2073-4360/11/5/752/s1. Figure S1: A) UV/vis spectra of TA in water (black, 0.001 mg/mL) and TA-Br in acetonitrile (ACN) (blue, 0.001 mg/mL). Different solvents were used due to the chemical solubilities. TA is insoluble in ACN and TA-Br is insoluble in water. Figure S2: The high resolution XPS Br 3d spectra of A) Tannic acid and B) TA-Br. Table S1: The polymer stars prepared by growing either or PMMA or P(OEO300MA) arms by photo ATRP (5.2 mW/cm2; λ= 365 nm). Figure S3: A) The GPC traces of TA-PMMA10 and B) TA-P(OEO300MA)37 before (black) and after (blue) degradation. Scheme S1: The synthesis of TP-iBBr. Figure S4: A) The GPC traces of TA-P(OEO300MA)25 degradation in sat. NaHCO3/MeOH. B) The UV/vis spectra over time. The solution prepared was 5 mg/mL TA-P(OEO300MA)25 (total solution volume 15 mL). Figure S5: The percent viability of (A) HEK293 cells and (B) HaCaT cells, after 72 h of TA-P(OEO300MA) polymer stars, samples after degradation, and OEO300MA monomer at four concentrations from 250μg-3 mg/mL. Positive (orange) and negative controls (Saponin, grey) are also shown.
