# Supplementary material for: Degradable Polymer Stars Based on Tannic Acid Cores by ATRP
Source: Polymers (Basel). 2019 Apr 28;11(5):752. doi: 10.3390/polym11050752 (PMC6571670; doi:10.3390/polym11050752)
Supplement: Supplementary file 1 [file polymers-11-00752-s001.pdf]

polymers

Communication

Supporting Information: Degradable Polymer Stars Based on Tannic Acid Cores by ATRP

Julia Cuthbert [1], Saigopalakrishna S. Yerneni[2], Mingkang Sun[1], Travis Fu[1], and Krzysztof Matyjaszewski [1,*]

[1] Department of Chemistry, Carnegie Mellon University, 4400 Fifth Avenue, Pittsburgh, Pennsylvania, 15213, United States; jcuthber@andrew.cmu.edu (J.C.); mingkang@cmu.edu (M.S.); travisfu@cmu.edu (T.F.)
[2] Department of Biomedical Engineering, Carnegie Mellon University, 5000 Forbes Avenue, Pittsburgh, Pennsylvania, 15213, United States; syerneni@andrew.cmu.edu (S.S.Y.)
* Correspondence: matyjaszewski@cmu.edu (K.M.)

Received: date; Accepted: date; Published: date

1. UV/vis Spectra

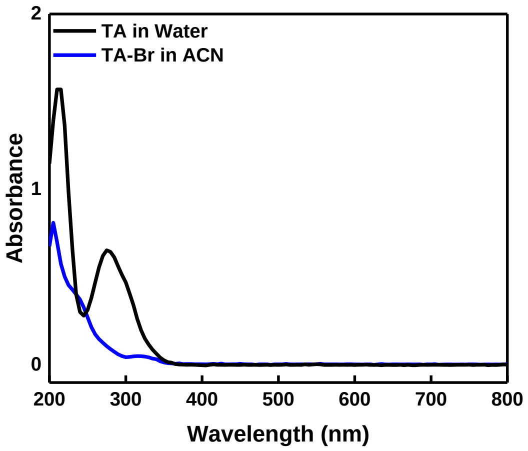

Figure S1. UV/vis spectra of tannic acid (TA) in water (black, 0.001 mg/mL) and TA-Br in acetonitrile (ACN) (blue, 0.001 mg/mL). Different solvents were used due to the chemical solubilities. TA is insoluble in ACN and TA-Br is insoluble in water.

2. XPS Br3d Scan

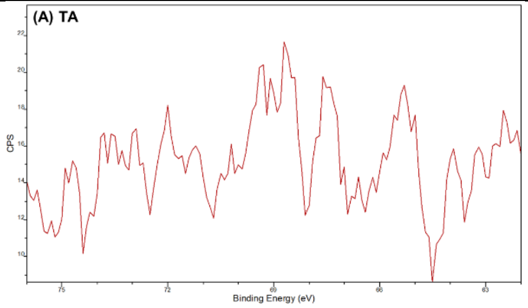

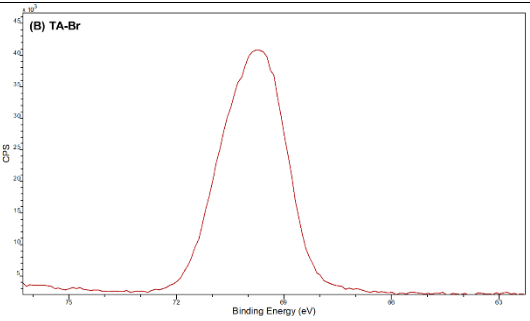

Figure S2. The high resolution XPS Br 3d spectra of **(A)** Tannic acid and **(B)** TA-Br.

3. Polymer Star Synthesis and Degradation

3.1. The polymer star synthesis

Table S1. The polymer stars prepared by growing either poly(methyl methacrylate) (PMMA) or P(OEO_{300}MA) arms by photo atom transfer radical polymerization (ATRP) (5.2 mW/cm2; λ= 365 nm).

Entry[1]	Core	Monomer	[M]/[Core]/[$CuBr_2$]/[Me_6TREN][1]	DP_{target}/arm[2]	Conv. (%)[3]
1	TA-Br	MMA	250/25/1/6	10	72
2	TA-Br	MMA	1250/25/1/6	50	50
3	TP-iBBr	MMA	2000/1/0.24/1.44	333	16
4	TA-Br	OEO_{300}MA	1500/25/1/6	60	43
5	TA-Br	OEO_{300}MA	5000/25/1/4	200	19

[1]Molar equivalents; [M] = monomer; Concentration: [MMA] = 0.9M; [OEO_{300}MA] = 0.5M; [2]Assuming 25 arms per star for TA-Br and 6 arms per star for TP-iBBr. [3]Determined by ^{1}H NMR conversion of vinyl bonds.

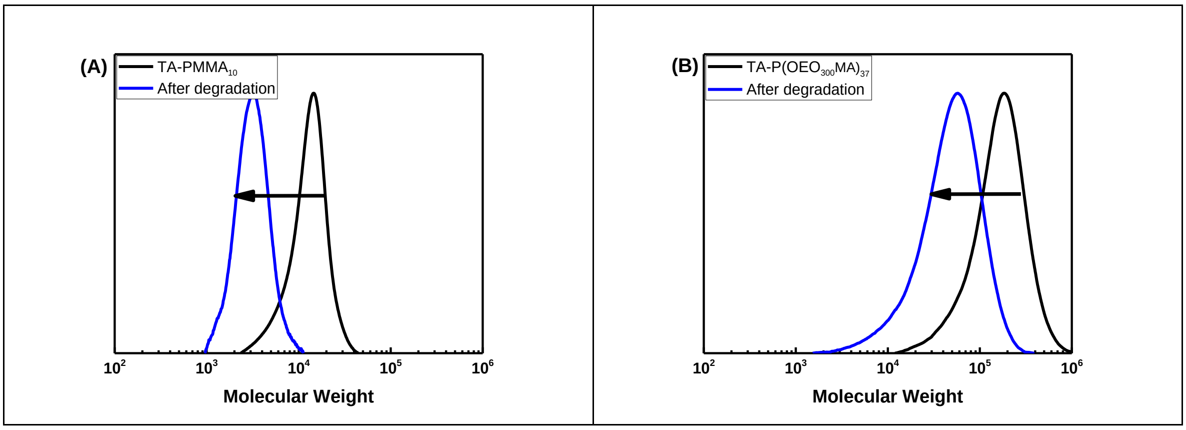

Figure S3. (A) The GPC traces of TA-$PMMA_{10}$ and **(B)** TA-P(OEO_{300}MA)$_{37}$ before (black) and after (blue) degradation.

3.2. TP-iBBr Synthesis

Scheme S1. The synthesis of TP-iBBr.

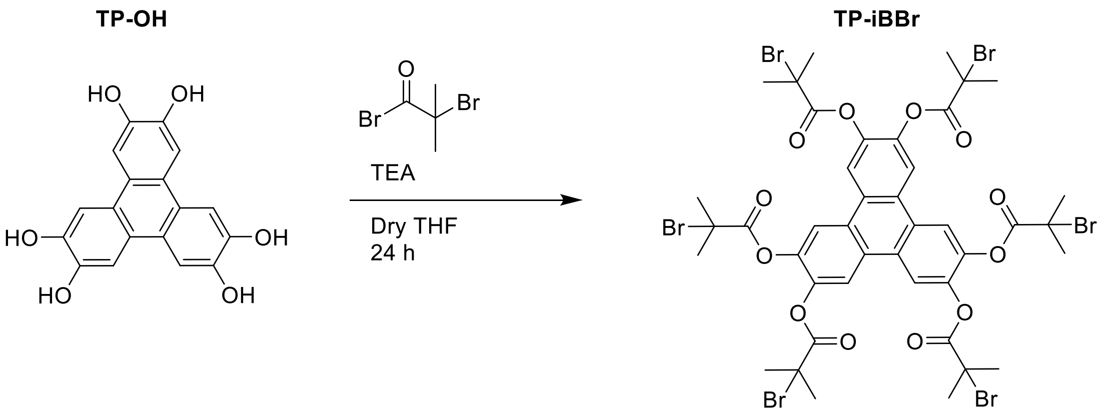

In a dry Schlenk flask (100 mL), TP-OH (1.2g, 3.70 mmol, 1 eq.) was dissolved dry THF (50 mL). TEA was added (4.3 mL, 30.8 mmol, 8.3 eq.) and the solution was cooled to 0°C in an ice bath. α-bromoisobutyryl bromide (3.58 mL, 30.0 mmol, 7.8 eq.) was added dropwise. Then the solution was removed from the ice bath and stirred at room temperature in the sealed flask overnight. The reaction mixture was gravity filtered and the solution was concentrated in vacuo. The solution was dissolved ether (200 mL), washed three times with $NaHCO_3$ saturated DI water solution (3x200 mL) and twice with distillated water (2x50 mL). The organic phase was concentrated in vacuo and dissolved in a minimum of ethyl acetate. The product (TP-iBBr) was obtained by precipitate in hexane and subsequent centrifugation (2.6 g, 2.13 mmol, molar mass = 1218.21 g/mol, yield = 58 %). ^{1}H-NMR: (500 MHz, $CDCl_3$) δ 8.31 (s, 6H), 2.16 (s, 36H).

3.3. Degradation followed by GPC and UV/vis

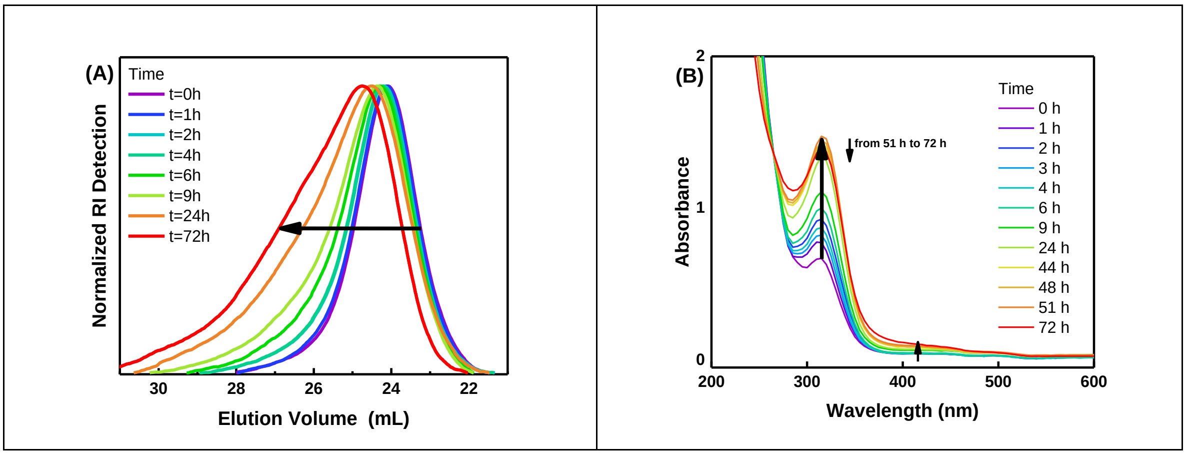

Figure S4. (A) The GPC traces of TA-P(OEO$_{300}$MA)$_{25}$ degradation in sat. $NaHCO_3$/MeOH. **(B)** The UV/vis spectra over time. The solution prepared was 5 mg/mL TA-P(OEO$_{300}$MA)$_{25}$ (total solution volume 15 mL). This solution was more concentrated in order to remove 1 mL samples at each time interval and have enough polymer for GPC.

4. Cytotoxicity Assays

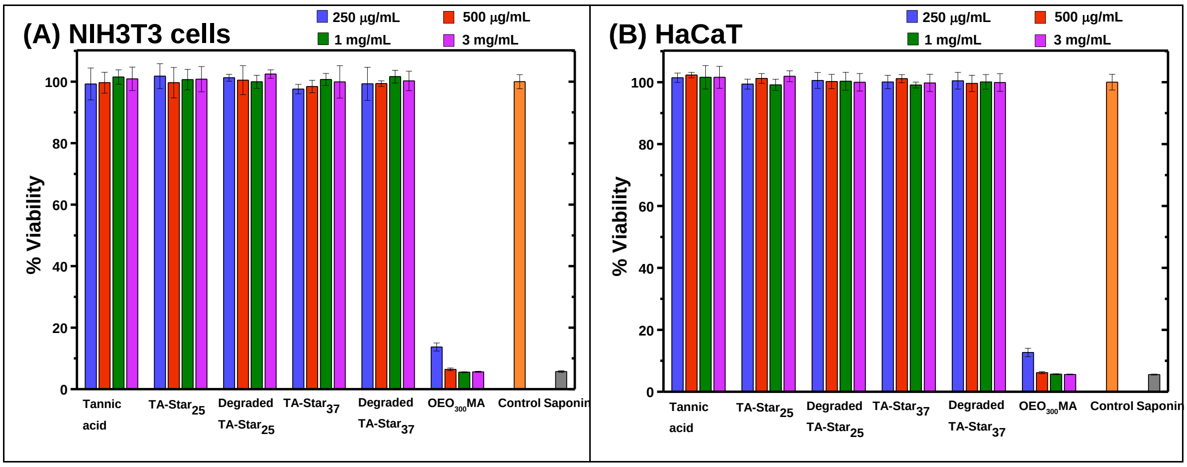

Figure S5. The percent viability of (A) NIH3T3 cells and (B) HaCaT cells, after 72 h of TA-P(OEO300MA) polymer stars, samples after degradation, and OEO300MA monomer at four concentrations from 250µg-3 mg/mL. Positive (orange) and negative controls (Saponin, grey) are also shown.
